# miR-373 promotes invasion and metastasis of colorectal cancer cells via activating ERK/MAPK pathway

**DOI:** 10.1038/s41598-023-49565-5

**Published:** 2024-01-02

**Authors:** Qian Chen, Yunfeng Li, Tailiang Lu, Jihui Luo, Li Yang, Zheng Zhou, Zeyu Tian, Siwen Tan, Qi Liu

**Affiliations:** 1 Department of Surgery, Hunan Provincial People’s Hospital, The First Affiliated Hospital of Hunan Normal University, Changsha, 410005 Hunan People’s Republic of China; 2 Department of General Surgery, Hunan Provincial People’s Hospital, The First Affiliated Hospital of Hunan Normal University, Changsha, 410005 Hunan People’s Republic of China; 3 Department of comprehensive Surgery, Hunan Provincial People’s Hospital, The First Affiliated Hospital of Hunan Normal University, Changsha, 410005 Hunan People’s Republic of China; 4 Department of Gastroenterology, Hunan Provincial People’s Hospital, The First Affiliated Hospital of Hunan Normal University, Changsha, 410005 Hunan People’s Republic of China; 5 Department of General Surgery, Hunan Provincial People’s Hospital, The First Affiliated Hospital of Hunan Normal University, No. 61 Jiefang West Road, Changsha, 410000 Hunan People’s Republic of China

**Keywords:** Cancer, Cell biology, Molecular biology, Oncology

## Abstract

To explore the relationship between miR-373 and the occurrence and development of colorectal cancer. Additionally, it aims to predict the potential cellular signaling pathways and regulatory mechanisms in which miR-373 may be involved and provides a theoretical basis and experimental evidence for the clinical application of miR-373 as a potential biomarker, molecular target, and prognostic indicator in colorectal cancer. Real-time quantitative PCR is used to analyze the expression of miR-373 in human colorectal cancer cell lines and normal human colonic epithelial cells. Further validation of the differential expression of miR-373 in colorectal cancer cell lines is being performed. Biological functions such as cell proliferation, invasion and apoptosis are being detected by MTT, CCK-8, transwell, cell cycle analysis, and flow cytometry experiments to verify the changes in the biological behavior of colon cancer cells after overexpression and interference of miR-373 in SW-480 cells and to explore the effects of miR-373 on cell proliferation, invasion, and apoptosis in colon cancer cells. Proteomic analysis is being conducted on proteins extracted from miR-373 overexpressing SW480 cells, and mass spectrometry is used for protein identification. GO, KEGG, and enrichment analysis are being employed to analyze the significantly differentially expressed proteins. The expression levels of pathway-related proteins are being verified using Western blot. Overexpression of miR-373 increased the invasive and metastatic ability of SW-480 cells; knockdown of miR-373 decreased the invasive and metastatic ability of SW-480 cells. However, there was no statistically significant effect on cell proliferation and apoptosis in SW-480 cells. Proteomic analysis identified 78 differentially expressed proteins based on fold change (FC) > 1.2 and P < 0.05. Annotation of differentially changed proteins revealed that the MAPK signaling pathway, PI3K-Akt signaling pathway, and FAK signaling pathway may play crucial roles in the migration and invasion of colorectal cancer. Western blot analysis showed that overexpression of miR-373 significantly increased the levels of p-ERK1/2 in SW480 cells. miR-373 may activate the ERK/MAPK signaling pathway to promote the invasion and migration of colorectal cancer cells.

## Introduction

Colorectal cancer (CRC), a common malignant tumor that poses a threat to human life and health, has shown an increasing incidence and mortality rate in China. According to the latest national cancer statistics released by the National Cancer Center in February 2022, there were 4.57 million new cases of cancer in China in 2020, with colorectal cancer accounting for 560,000 cases, ranking second among all new cancer cases, second only to lung cancer. In terms of cancer-related deaths, there were 3 million deaths in China in 2020, with colorectal cancer accounting for 290,000 deaths, ranking fifth^1^. Despite advances in comprehensive treatment methods, including surgery supplemented with chemotherapy, targeted therapy, and other approaches, the prognosis for patients with advanced colorectal cancer has not significantly improved, with a 5-year survival rate remaining around 50–60%. Tumor metastasis is the primary cause of death in colorectal cancer patients, therefore, highlighting the importance of studying the mechanisms underlying tumor metastasis.

Currently, miR-373 has been confirmed to have differential expression in various malignant tumors and is closely related to cancer. For example, miR-373 is highly expressed in endometrial cancer tissues and its expression level is related to the malignancy of endometrial cancer^2^. It is considered as a tumor suppressor gene that inhibits the proliferation and invasion of breast cancer cells by regulating the target gene Bcl-6^3^. The expression of miR-373-3p is associated with the overall survival rate of prostate cancer patients, and patients with longer survival have higher expression levels of miR-373-3p. Therefore, miR-373-3p is considered the best choice for early diagnostic markers to distinguish prostate cancer from benign prostatic hyperplasia^4^. In addition, the expression of miR-373 is decreased in pancreatic adenocarcinoma^5^, while it is increased in gastric adenocarcinoma^6^, esophageal cancer^7^, and liver cancer^8^, indicating that miR-373 may play a role as an oncogene or tumor suppressor gene in different malignant tumors. The role of miR-373 in colorectal cancer has not been consistent in different studies. Wang^9^ found that miR-372 and miR-373 enhance the stemness of colorectal cancer cells by inhibiting the expression of differentiation genes such as NFkB, MAPK/Erk, and vitamin D receptor (VDR). The expression levels of miR-143 and miR-145 are decreased in colorectal cancer^10,11^. In apoptotic cells, the expression level of miR-143 gradually increases with the progression of apoptosis, while it significantly decreases during the cell proliferation phase^12^. Transfection of miR-143 precursor in DLD-1 and SW-480 cells confirms that the upregulation of miR-143 inhibits cell proliferation, indicating that miR-143 suppresses the proliferation of colon cancer cells. According to Zhang^13^, there is a certain correlation between stage IV colon cancer and the expression level of miR-141, and further research suggests that higher expression levels of miR-141 are associated with lower overall survival rates, indicating that miR-141 can be used to predict prognosis in colon cancer.

In general, the research on miRNA in colorectal cancer is still quite limited. There are even fewer studies on its related proteomic research in the direction of colorectal cancer. Based on the differential carcinogenic or anti-cancer effects of its expression in other malignant tumors, and the unclear carcinogenic or anti-cancer mechanisms, more experiments are needed to verify its relationship with the occurrence and development of malignant tumors to further determine its application value. In order to further study the correlation between the expression of miRNA in colorectal cancer and the biological behavior of colorectal cancer cells, and to find its possible mode of action, differential expression of miR-373 was discovered in colorectal cancer tissues and adjacent normal tissues through the TCGA database. miR-373 is a member of the miR-520/373 family, and its seed sequences are different in each species. For example, the miR-290-295 cluster in mice is homologous to the miR-371-373 cluster in humans^14^. There is limited research on miR-373 in colorectal cancer, but the complex functions of miRNA suggest that the gene expression regulation mediated by miR-373 may have broader and more profound significance in cancer research, and it can serve as a biomarker for cancer-related diagnosis, treatment, and prognosis assessment. According to the NCBI Entrez Gene, the nucleotide sequence is GGGAUACUCAAAAUGGGGGCGCUUUCCUUUUUGUCUGUACUGGGAAGUGCUUCGAUUUUGGGGUGUCCC, and the hsa-miR-373-3p sequence is GAAGUGCUUCGAUUUUGGGGUGU. Therefore, this study explores the impact of abnormal expression of miR-373 on the biological behavior of colorectal cancer, further investigates the relationship between miR-373 and the occurrence and development of colorectal cancer, uses proteomic methods to study the protein expression characteristics of human colon cancer SW480 cells under miR-373 overexpression, predicts the cell signaling pathways and regulatory mechanisms that miR-373 may be involved in, and evaluates the predictive value of miR-373 as a potential monitoring marker for colorectal cancer invasion and metastasis.

## Methods

### Cell lines and culture

Colorectal cancer cell lines (HCT116, CACO-2, SW480, LOVO) and normal human colonic epithelial cell line (Hcopic) were obtained from the Chinese Academy of Sciences (China, Shanghai) and stored in the Experimental Laboratory of Hunan Cancer Hospital. All cells were cultured in DMEM medium supplemented with 10% FBS and incubated in CO_2_ incubator at 37 °C with 5% CO_2_.

### RNA isolation and qRT-PCR

The total RNA was isolated using Trizol reagent (Pufei, Shanghai) according to the manufacturer’s protocol. The RNA was reverse transcribed into cDNA using the Promega M-MLV kit (Takara, China). All samples were analyzed using SYBR Green PCR Master Mixture (Takara, China). U6 expression was acted as internal loading control for normalization, miR-373 relative gene expression determinations were made with the comparative delta–delta CT method (2–ΔΔCt). The reaction mixtures of U6 and miR-373 were incubated at thermal cycling conditions comprised 95 ℃ for 30 s, and 40 cycles at 95 ℃ for 5 s followed by 60 ℃ for 30 s.

### The construction, infection, and validation of overexpression and knockdown miR-373 viruses

Overexpression and knockdown of miR-373 viruses were constructed by GeneChem (Shanghai, China). The purpose viruses were infected into SW480 cells and selected by puromycin, and then divided into four groups for continuous cultivation. After 72 h, fluorescence observation was conducted under a microscope. If the cell infection efficiency reached above 80%, they could be used for subsequent experiments. The groups included: NC-KD (control group, SW480 cells cultured normally, negative control CON313 lentiviral infection group); KD (experimental group, SW480 cells cultured normally, LV-hsa-miR-373-3p-inhibition lentiviral infection group); NC-OE (control group, SW480 cells cultured normally, negative control CON238 lentiviral infection group); OE (experimental group, SW480 cells cultured normally, LV-hsa-mir-373 lentiviral infection group).

### Cell proliferation assay

For MTT assays, SW480 cells were seeded in a 96-well plate at a density of 2000 cells per well. The cells were infected with overexpressing or interfering miR-373 viruses as described above. MTT assays were performed at designated time points. The cells were incubated with 20 μl of 0.5% MTT at 37 ℃ for 4 h. The color produced by the cells was incubated in 100 μL of DMSO and the absorbance was measured at 490 nm using an ELISA reader to record OD values. Relative cell growth was determined by the ratio of the average absorbance of treated cells to the average absorbance of control cells.

To assess cell viability, 10 μL of CCK-8 (BestBio, China) was added to each well at 0, 24, 48, and 72 h after transfection and incubated for 4 h. The absorbance was measured at 450 nm using a Microplate Reader (Thermo Scientific).

### Cell migration and invasion assays

Transwell chambers (Corning, NY, USA) were used, with the upper chamber equipped with an 8-μm-pore polycarbonate filter pre-coated with Matrigel. In the cell migration assay, a total of 100,000 cells were cultured in each upper chamber using serum-free media (Corning). Meanwhile, the lower chamber was filled with media supplemented with 10% fetal bovine serum. After incubating at 37 °C for 24 h, the cells in the upper chamber were removed using a cotton swab. Then, the lower part of the chamber was fixed with 4% PFA for 30 min and stained with 0.1% crystal violet for observation and photography. The migrated cells were counted, and statistical analysis.

### Flow cytometry analysis for cell cycle and apoptosis

For cell apoptosis detection, cells were digested with trypsin, resuspended in cell suspension, and centrifuged at 1300 *rpm* for 5 min to collect cells. The cells were then resuspended in binding buffer and stained with 10 μl Annexin V-APC. After incubating in the dark at 37 °C for at least 30 min, cell apoptosis rate was analyzed using flow cytometry (BD Biosciences).

For cell cycle detection, cells were digested with trypsin, washed with pre-chilled DPBS (pH = 7.2–7.4) at 4 °C, and centrifuged at 1300 *rpm* for 5 min to collect cells. The cells were then fixed with PBS, washed three times, and suspended in PBS containing 10 μg/ml propidium iodide and 100 μg/ml RNaseA. After incubating in the dark at 37 °C for at least 30 min, cell cycle was analyzed using flow cytometry. The data was analyzed using ModFit software to determine the percentage of cell apoptosis.

### Overexpressed miR-373 identification by differential protein profiling in SW480 cells

The TMT quantitative proteomic analysis was performed using a high-resolution mass spectrometer, Q Exactive HF-X. The resulting raw spectral files were converted into a suitable format using Proteome Discoverer 2.2 software and submitted to the MASCOT 2.6 server for database searching. Qualitative analysis of the mass spectrometry data was conducted using the Homo sapiens sub-database from the Uniprot database. The search results generated by Proteome Discoverer 2.2 on the MASCOT server were filtered for an FDR < 0.01 to obtain reliable qualitative results.

### GO function and enrichment analysis

The GO function and enrichment analysis of differentially expressed proteins were performed using Blast2Go (https://www.blast2go.com/). The annotation results were visualized using the ggplot2 package to create a bar graph. The number of differentially expressed proteins was counted at the GO level 2 functional annotation level. The significantly different proteins identified in the experiment were compared to the total protein dataset based on GO annotation results, and the significant differences between the two groups were determined using Fisher's enrichment test (P < 0.05).

### KEGG pathway and enrichment analysis

KOALA (KEGG Orthology And Links Annotation, V2.2) is applied to align the target protein sequences with the KEGG GENES database, automatically obtaining pathway information involved through KO classification, and ggplot2 package is used to generate bar charts. Using KEGG pathways as units and all qualitative proteins as background, Fisher's exact test is used to analyze and calculate the significance level of protein enrichment in each pathway.

### Protein isolation and western-blot analysis

Total protein extracts were obtained from tissues and cells using RIPA lysis buffer. The protein concentration was then quantified using the BCA Protein Assay Kit (Thermo, USA). The protein samples were separated on SDS-PAGE and transferred onto PVDF membranes. After blocking with 5% skim milk for 4 h, they were incubated with primary antibodies while gently shaking at 4 ℃ overnight, followed by incubation with secondary antibodies. Finally, the protein blots were visualized using the ECL kit (cell signaling technology).

### Statistical analysis

Each experiment in this study was repeated three times. All data quantification and statistical analysis were performed using SPSS 26.0 software. Means ± SD were used to express the normal measurement data. Differences between groups were calculated using the t-test or one-way ANOVA. Graphs were generated using Prism 9.0 software. A difference with a P value < 0.05 was considered statistically significant.

### Ethical approval

This study was approved by the Ethics Committee of The First Affiliated Hospital of Hunan Normal University (The Hunan Provincial People’s hospital).

## Results

### Expression of miR-373 in Hcopic and CRC cell lines

**Figure 1:**
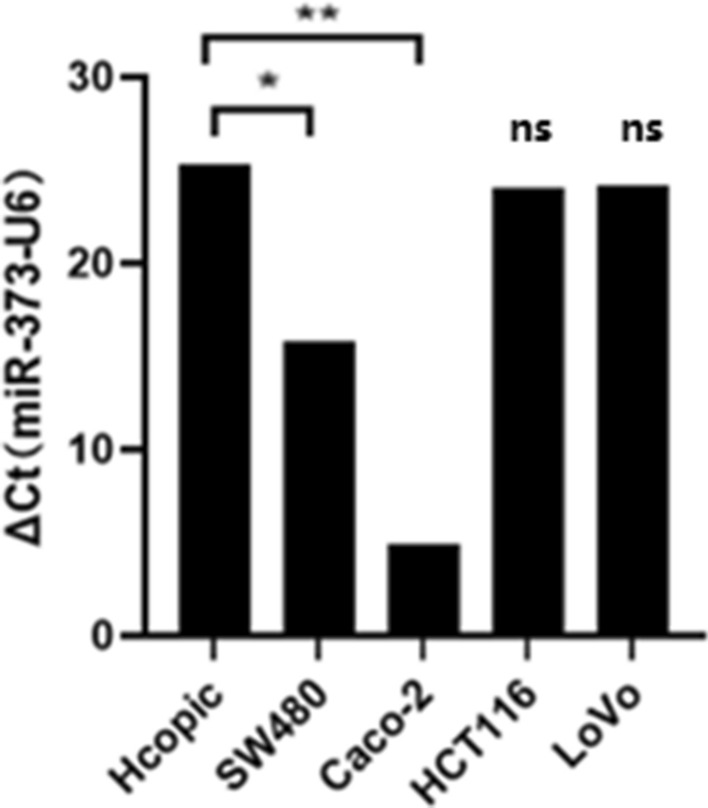
The expression of miR-373 in Hcopic and CRC cell lines. The expression level of miR-373 was upregulated by RT-qPCR in colorectal cancer cells. *p < 0.05, **p < 0.01.

To confirm the involvement of miR-373 in the occurrence and development of colorectal cancer, we used real-time fluorescence quantitative qRT-PCR method to detect the expression level of miR-373 in four colorectal cancer cell lines (HCT-116, SW-480, Lovo, Caco-2) and normal colonic epithelial cell line (Hcopic). The expression abundance of miR-373 was highest in Caco-2 cells, moderate in SW-480 cells, and significantly different from the expression level in Hcopic. There was no significant difference in miR-373 expression level among Hcopic, Lovo, and HCT-116 cell lines (Fig. 1).

### Stable transfection efficiency of overexpression and interference of miR-373 virus

**Figure 2:**
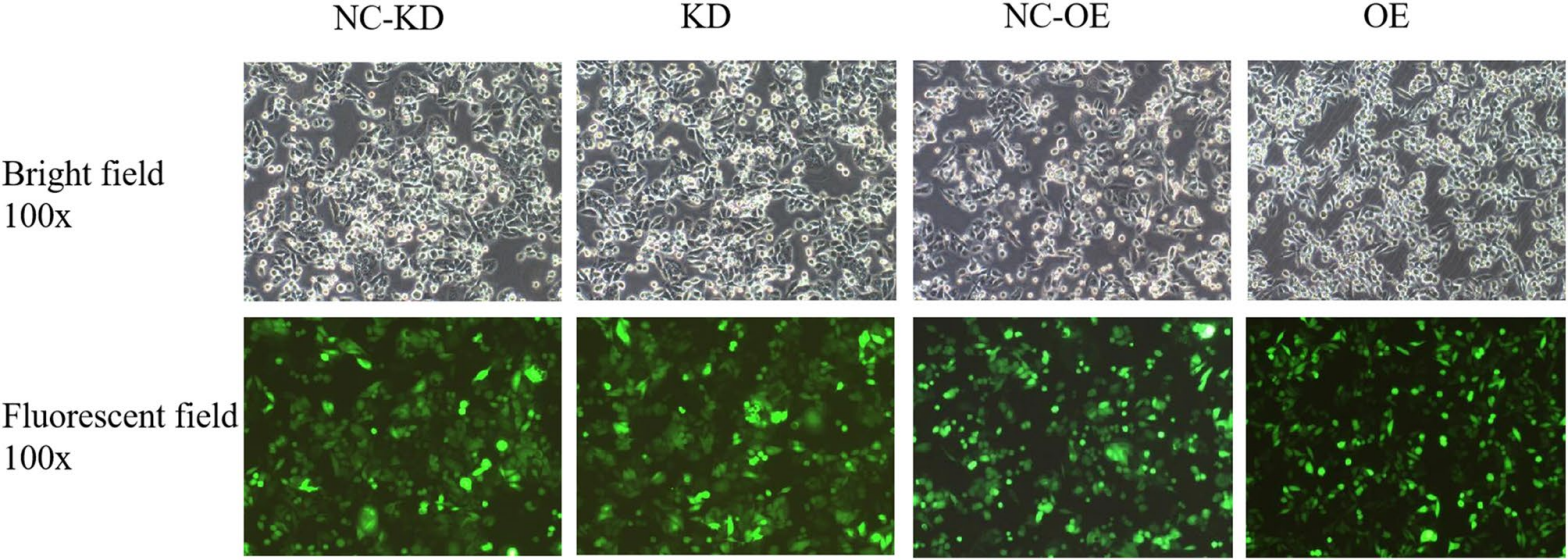
The fluorescence observation under a microscope after transfection with viruses for 72 h. After transfection with viruses according to the experimental design, the fluorescence of cells was observed under a fluorescence microscope, and the fluorescence intensity accounted for more than 80% of the total cell count. *NC-KD* miR-373 interference negative control group, *KD* miR-373 interference group, *NC*-*OE* miR-373 overexpression negative control group, *OE* miR-373 overexpression group.

**Figure 3:**
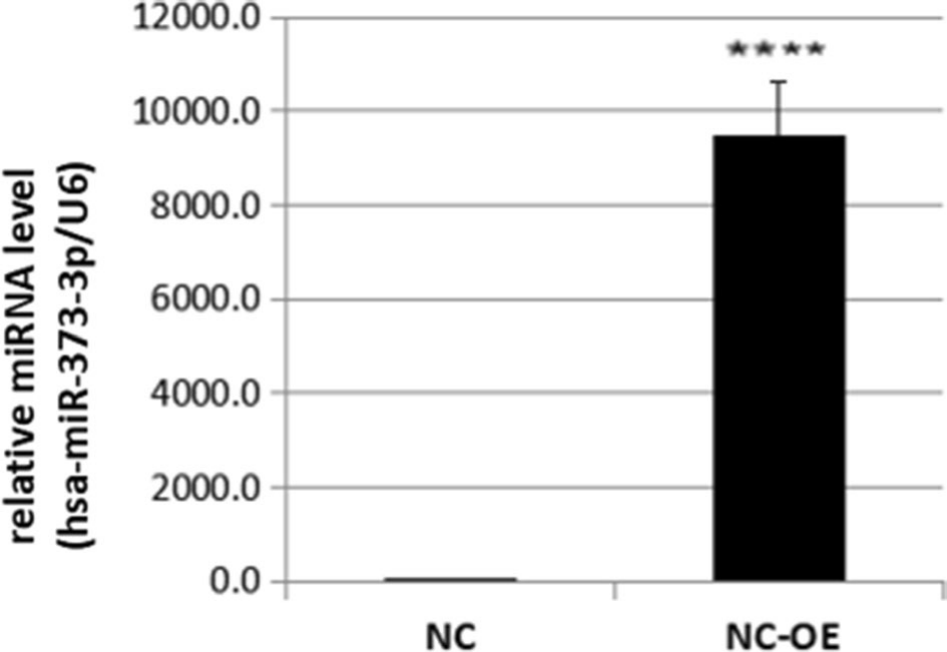
The expression level of miR-373 in SW-480 overexpression cells compared to the negative control group. There was a significant difference in the expression level of miR-373 between SW-480 cells transfected with overexpression viruses (NC-OE) and the overexpression negative control group (NC) (p < 0.05).

SW480 cells were selected for transfection, and after transfection of miR-373 overexpression and interference viruses respectively, the target virus-infected SW480 cells were observed under a fluorescence microscope after 72 h. The results showed that the cell infection efficiency was ≥ 80%, indicating that subsequent experiments could be conducted (Fig. 2). After transfection with miR-373 overexpression virus, q-PCR was used to detect the expression levels of miR-373 in the overexpression group and control group in SW-480 cells. The target virus successfully infected the target cells, and the overexpression negative control group (miR-373 NC-OE), overexpression group (miR-373 OE), interference negative control group (miR-373 NC-KD), and interference group (miR-373 KD) were successfully constructed, forming four experimental groups. q-PCR verified that there was a significant difference in the expression levels of miR-373 between the transfection of miR-373 overexpression virus and the overexpression negative control group (p < 0.05) (Fig. 3).

### miR-373 promoted colorectal cancer cell invasion and metastasis

**Figure 4:**
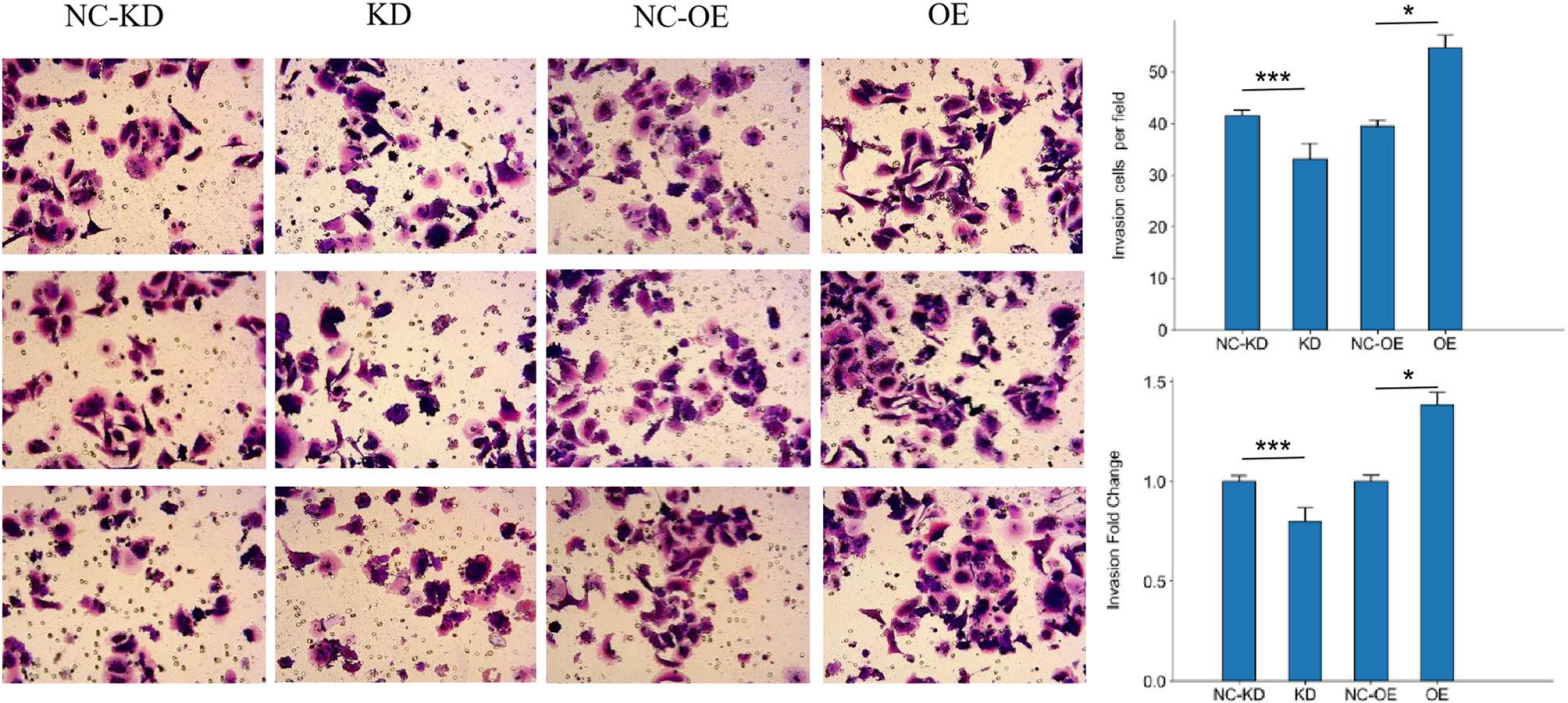
The effect of miR-373 on the invasive ability of SW480 cells. After 48 h, cell counting was performed under a microscope (200x) in each group, and the proportion and fold change of invasive cells in each experimental group are shown on the right. *p < 0.05, **p < 0.01, ***p < 0.001. *NC*-*KD* miR-373 interference negative control group, *KD* miR-373 interference group, *NC*-*OE* miR-373 overexpression negative control group, *OE* miR-373 overexpression group.

Invasion of the extracellular matrix is an important step in tumor metastasis, and the ability of tumor cells to penetrate the reconstructed basement membrane is closely related to their invasive and metastatic abilities in vivo. The invasion ability of colorectal cancer cells was determined by counting the number of cells entering the lower chamber. SW480 cells overexpressing miR-373 showed a significantly higher invasion and metastasis rate compared to the NC-KD group, as analyzed by T-Test (P < 0.05). The number of colorectal cancer cells invading the lower chamber was significantly increased in the miR-373 overexpression group compared to the control group. SW480 cells with miR-373 interference showed a significantly higher invasion and metastasis rate compared to the NC-OE group, as analyzed by T-Test (P < 0.05) (Fig. 4). These results indicate that overexpression of miR-373 enhances the invasion ability of colorectal cancer cells, indirectly suggesting that miR-373 may play a role in promoting cancer in colorectal cancer.

**Figure 5:**
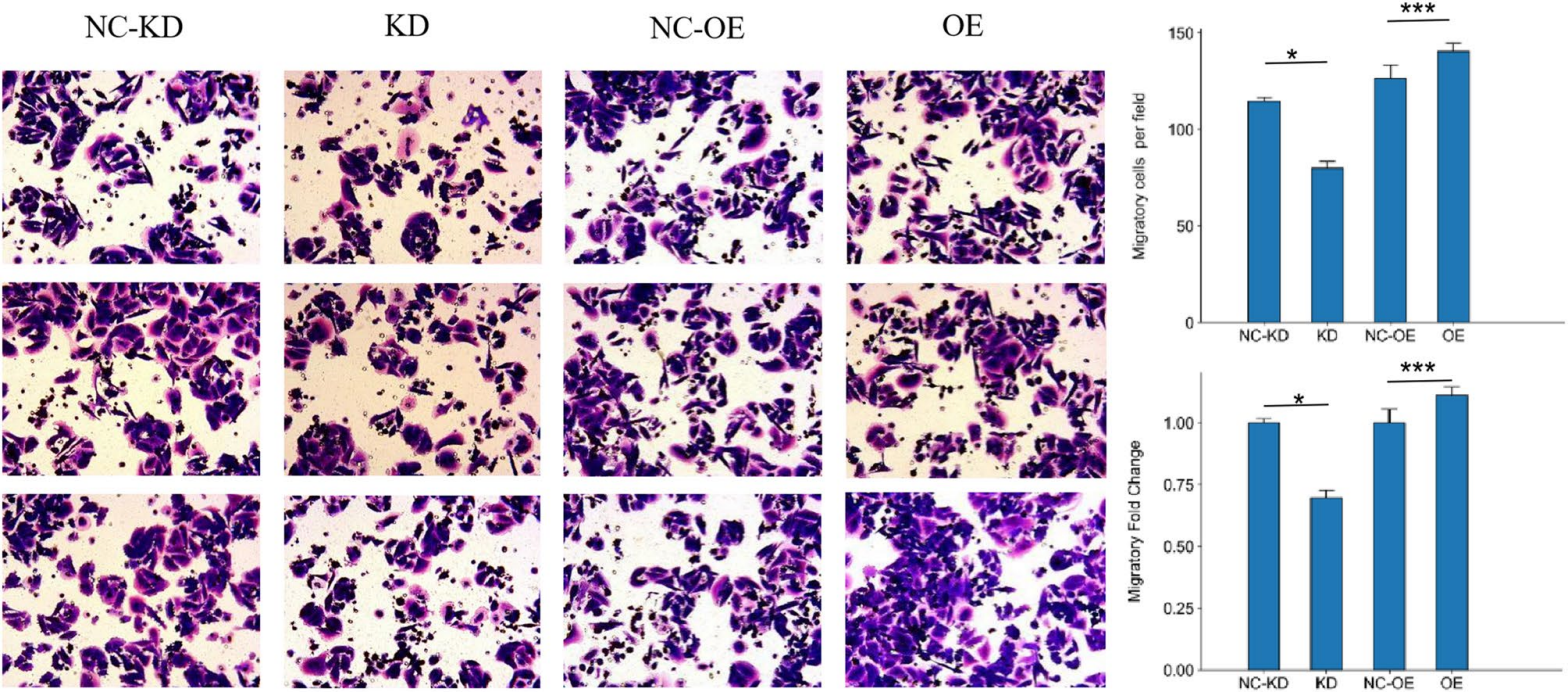
The effect of miR-373 on the migration of SW480 cells. Cell counting was performed under a microscope (200x) in each group after 48 h. *p < 0.05, **p < 0.01, ***p < 0.001. *NC*-*KD* miR-373 interference negative control group, *KD* miR-373 interference group, *NC*-*OE* miR-373 overexpression negative control group, *OE* miR-373 overexpression group.

Cell migration is an important indicator of tumor metastasis, and Transwell can be used to study the motility of colorectal cancer cells. SW480 cells overexpressing miR-373 showed a significantly higher Transwell migration rate compared to the NC-KD group, as analyzed by T-Test (P < 0.05). The number of colorectal cancer cells migrating into the lower chamber was significantly increased in the miR-373 overexpression group compared to the control group. SW480 cells with miR-373 interference showed a significantly higher Transwell migration rate compared to the NC-OE group, as analyzed by T-Test (P < 0.05) (Fig. 5). These results indicate that overexpression of miR-373 enhances the migration ability of colorectal cancer cells, indirectly suggesting that miR-373 may play a role in promoting cancer in colorectal cancer.

### miR-373 does not affect the proliferation and apoptosis, arrest cell cycle of colorectal cancer

**Figure 6:**
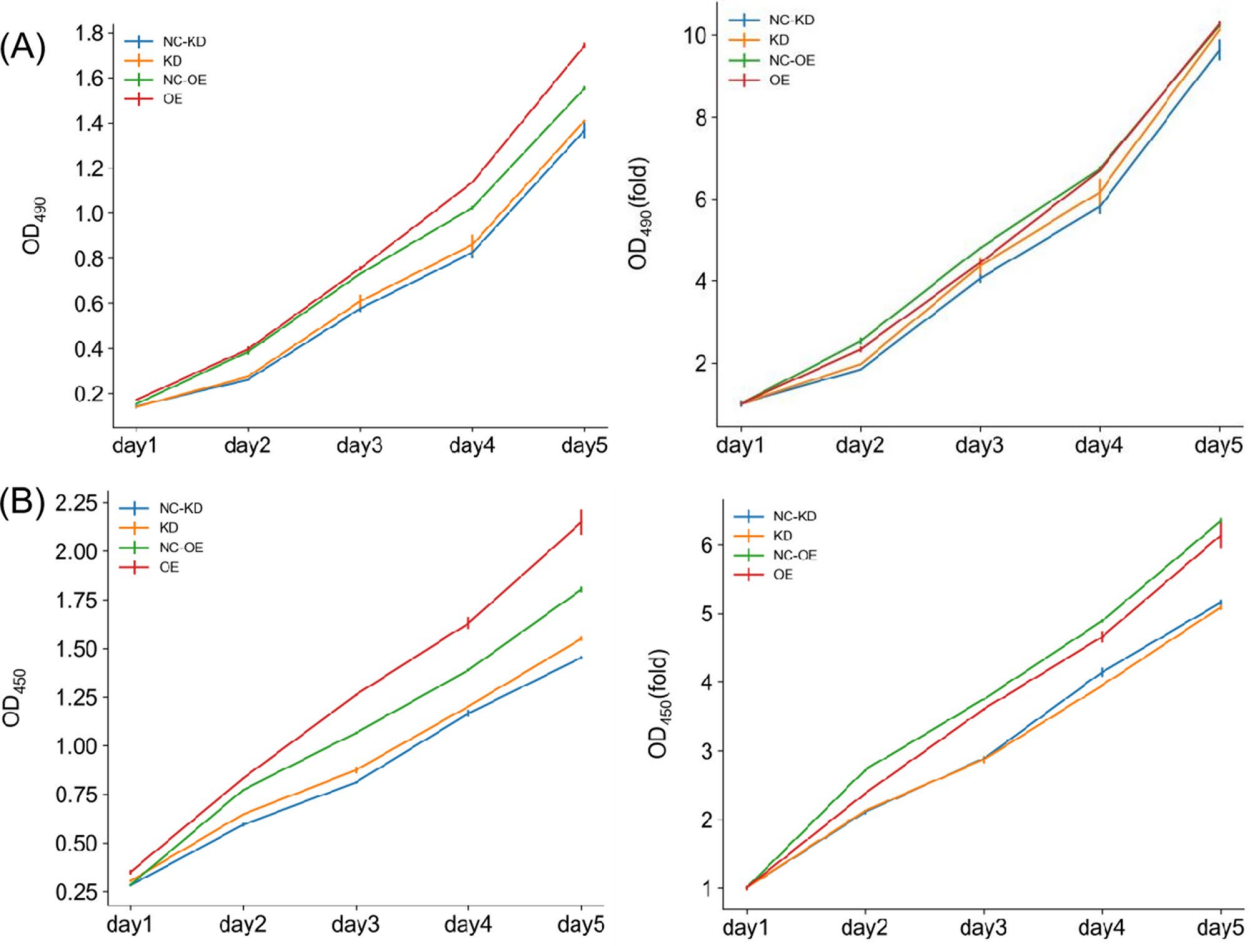
The effect of miR-373 on the proliferation of SW480 cells. Cell counting was performed in each experimental group from day 2 to day 5. *p < 0.05. (**A**) Absorbance at 490 nm, OD490/fold represents the fold change of OD490 in each experimental group relative to day 1, indicating the proliferation fold change on the corresponding day. (**B**) Absorbance at 450 nm, OD450/fold represents the fold change of OD450 in each experimental group relative to day 1, indicating the proliferation fold change on the corresponding day. The differences were not statistically significant (P < 0.05).

Tumor cells have the ability to proliferate rapidly and indefinitely. In our study, the MTT assay was used to detect cell viability, and the absorbance value reflecting the association between miR-373 and tumor cell proliferation was measured at a wavelength of 490 nm using an enzyme-linked immunosorbent assay (ELISA). The CCK8 assay was used to detect cell proliferation, and the absorbance value reflecting cell proliferation was measured at a wavelength of 450 nm using an ELISA. The results showed (Fig. 6): Compared with the NC-KD group, the SW480 cell line overexpressing miR-373 showed no statistically significant difference in cell proliferation based on T-Test analysis (P < 0.05); Compared with the NC-OE group, the SW480 cell line with miR-373 interference showed no statistically significant difference in cell proliferation based on T-Test analysis (P < 0.05). These results indicate that overexpression or interference of miR-373 does not significantly affect the proliferative ability of colorectal cancer cell lines, suggesting that miR-373 does not affect the unlimited proliferation of colorectal cancer cells.

**Figure 7:**
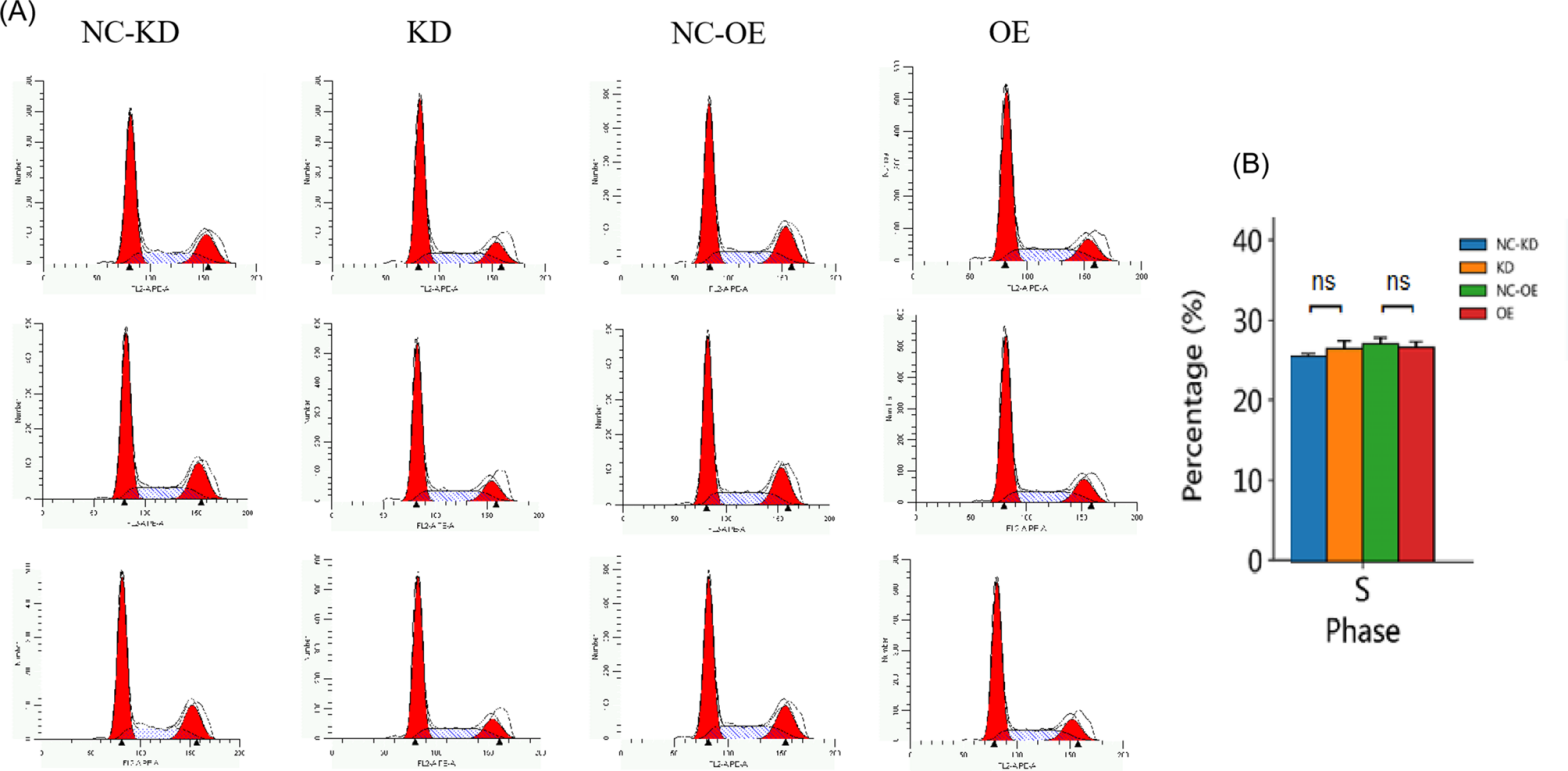
The role of miR-373 in the cell cycle of SW480 cells. (**A**) PI fluorescence intensity in each experimental group. (**B**) Bar chart showing the proportion of cells in the S phase of the cell cycle (vertical axis represents the percentage of cells in the S phase). Interference and overexpression of miR-373 had no effect on the S phase of the cell cycle in SW-480 cells (p > 0.05).

Flow cytometry was used to detect the fluorescence intensity of propidium iodide (PI), which directly reflects the DNA distribution status of cells at different phases of the cell cycle. The percentages of cells at each phase were calculated to reflect the effect of miR-373 on the cell cycle of colorectal cancer cells. The results showed (Fig. 7): Compared with the NC-KD group, the SW480 cell line overexpressing miR-373 showed statistically significant differences in cells at the G1 and G2/M phases based on T-Test analysis (P < 0.05), but no significant difference in cells at the S phase (P > 0.05); Compared with the NC-OE group, the SW480 cell line with miR-373 interference showed statistically significant differences in cells at the G1 and S phases based on T-Test analysis (P < 0.05), but no significant difference in cells at the G2/M phase (P > 0.05).

**Figure 8:**
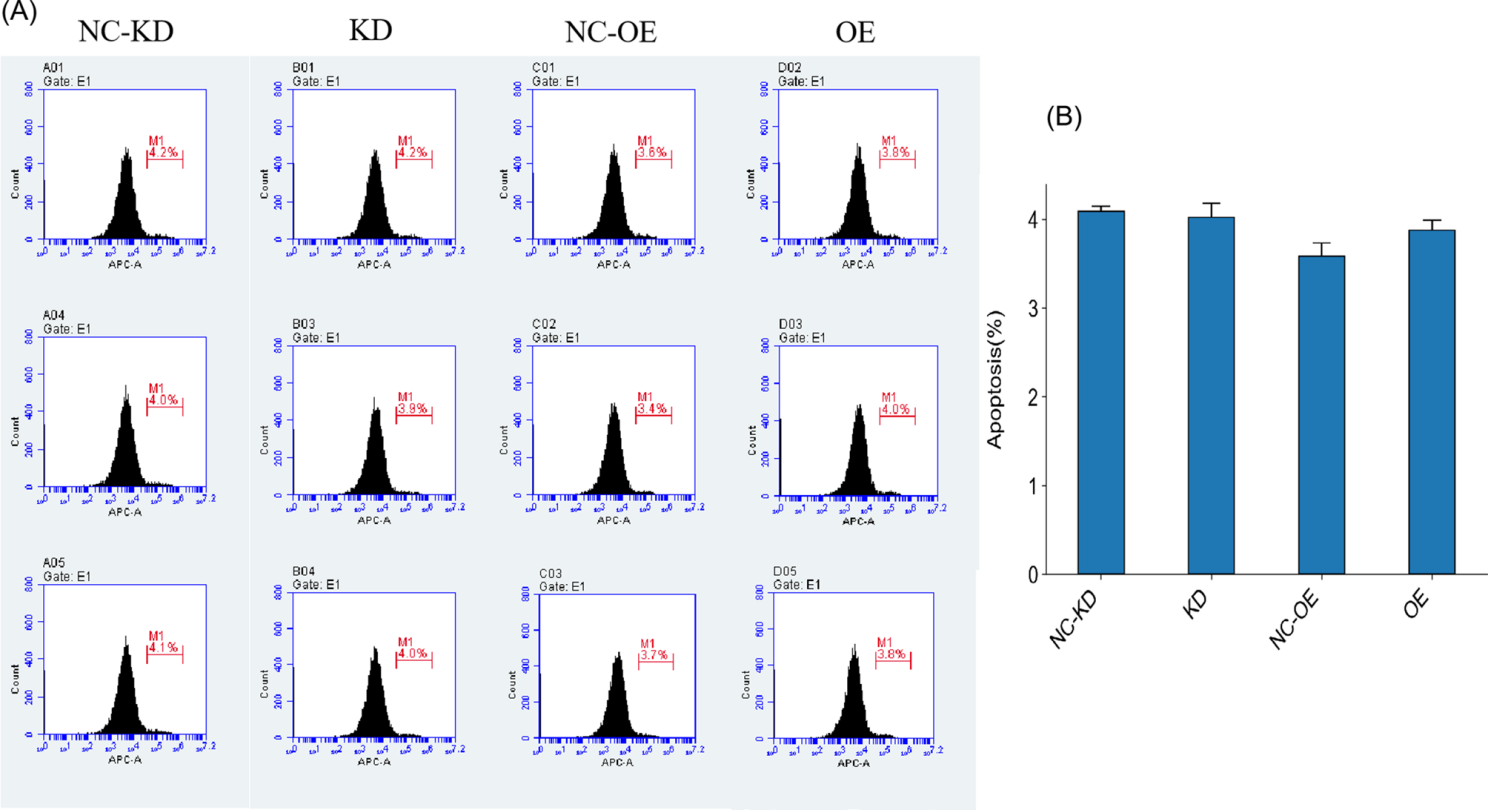
The effect of miR-373 on apoptosis of SW480 cells. (**A**) Flow cytometry apoptosis plots in each group. (**B**) Bar chart showing the proportion of apoptotic cells in each group. Interference and overexpression of miR-373 had no effect on cell apoptosis in SW-480 cells (p > 0.05).

Annexin V labeling with fluorescence was used to detect phosphatidylserine (PS) exposed on the cell membrane, thereby detecting cells in early apoptosis. The results showed (Fig. 8): Compared with the NC-KD group, the SW480 cell line overexpressing miR-373 showed no statistically significant difference in apoptotic cells based on T-Test analysis (P > 0.05); Compared with the NC-OE group, the SW480 cell line with miR-373 interference showed no statistically significant difference in apoptotic cells based on T-Test analysis (P > 0.05).

### miR-373 overexpression SW480 cell differential proteomic identification

#### Screening of differentially expressed proteins

**Figure 9:**
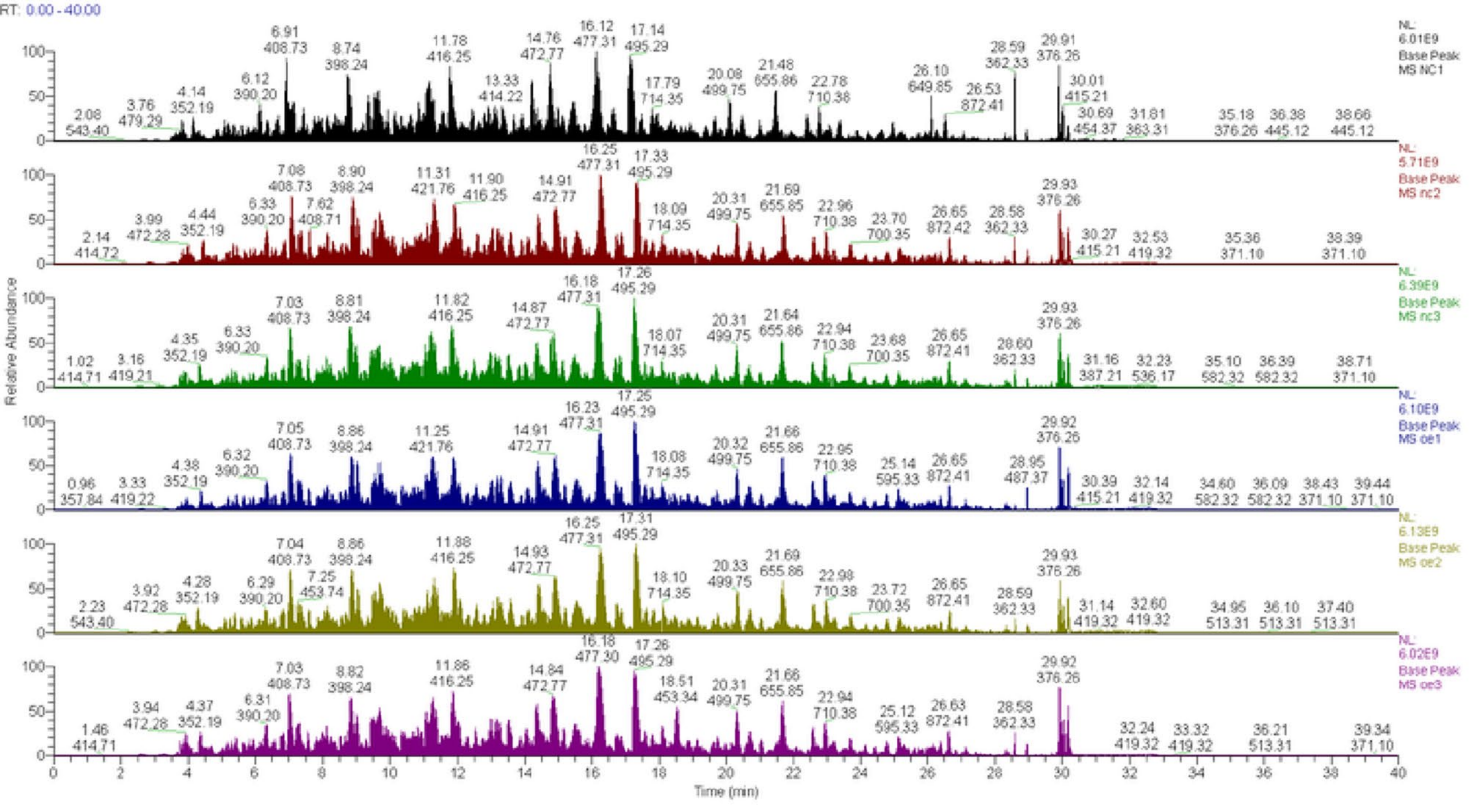
TMT quantitative proteomics analysis.

**Figure 10:**
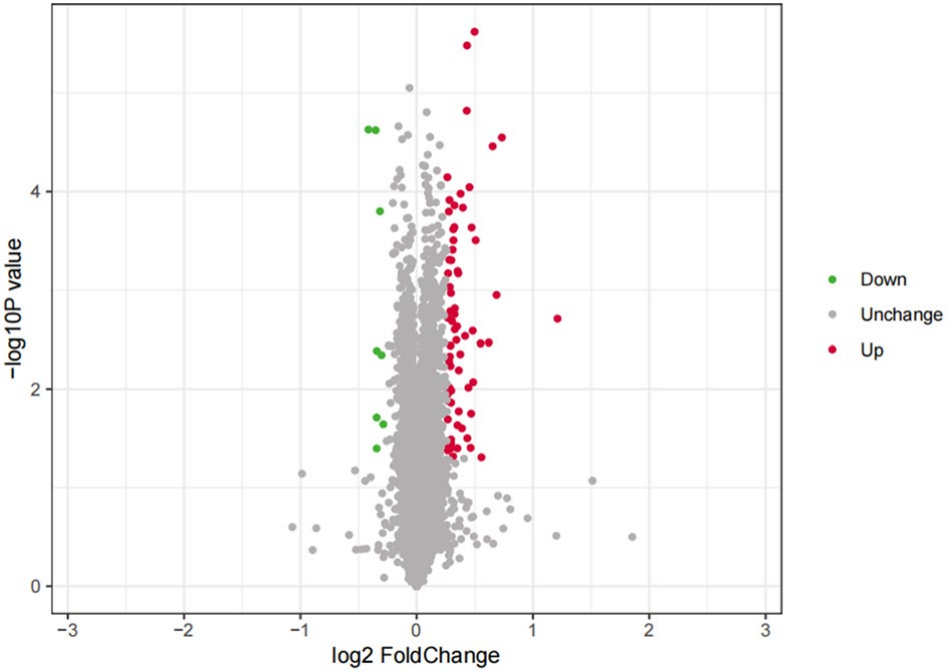
Shows the distribution of proteins in a volcano plot. In this plot, the x-axis is plotted with the log2-transformed fold change between the small sample and the normal sample, and the y-axis represents the − log10 transformed P-value of each protein (P < 0.05).

Using the high-resolution mass spectrometer Q Exactive HF-X for TMT quantitative proteomics analysis, we obtained the raw spectrum files (Fig. 9). A total of 539,881 spectra were obtained, with 125,187 valid spectra identified and 79,595 peptide segments identified using Mascot 2.6 and Proteome Discoverer 2.2. Among them, 74,469 were specific peptide segments, and a total of 8,178 proteins were quantified. After evaluating the significance of differences in the results using a T-test, 78 differentially expressed proteins with a p-value of less than 0.05 and a ratio > 1.2 were selected. The results showed that there were 70 significantly upregulated proteins and 8 significantly downregulated proteins (Table 1, Fig. 10). The volcano plot provides a visual and reasonable representation of the differentially expressed proteins (Fig. 10).

**Table 1:** Upregulated and downregulated proteins with the top tenfold change.

Accession	FC	R	P value	Accession	FC	R	P value
Q9BTV4	1.412143144	UP	2.38652E-06	P28360	0.750072908	DOWN	2.34629E-05
P80188	1.349647612	UP	3.29035E-06	P11169	0.782828283	DOWN	2.38279E-05
Q8NDM7	1.347945205	UP	1.51136E-05	P47929	0.80342651	DOWN	0.000158276
Q9Y2Y6	1.661934339	UP	2.82104E-05	Q14533	0.788375559	DOWN	0.004104687
Q96L50	1.572898799	UP	3.46669E-05	Q02410	0.811047389	DOWN	0.004520402
P02787	1.200586725	UP	7.15138E-05	Q9Y324	0.787607983	DOWN	0.019400036
O75385	1.369668246	UP	9.01842E-05	O43325	0.819532909	DOWN	0.022709099
P14210	1.298467433	UP	0.000105043	Q5W0U4	0.78784267	DOWN	0.03999535
O14933	1.215657312	UP	0.000121639				
P05362	1.252252252	UP	0.00013777

### GO function and enrichment analysis of differential proteins

**Figure 11:**
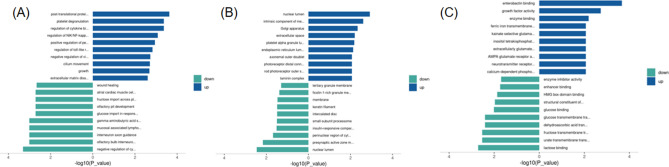
GO functional enrichment analysis of differentially expressed proteins. (**A**) Bar chart of enriched GO functions (BP) for upregulated and downregulated proteins. (**B**) Bar chart of enriched GO functions (CC) for upregulated and downregulated proteins. (**C**) Bar chart of enriched GO functions (MF) for upregulated and downregulated proteins. Vertical axis: significantly enriched GO terms from the analysis. Horizontal axis: − log10 transformed P-values. The longer the bar, the more significant the enrichment. The color of the bar represents the upregulation or downregulation of the protein.

We conducted GO enrichment analysis on significantly upregulated and downregulated proteins from three perspectives: biological processes, cellular functions, and molecular functions. The results showed that for significantly upregulated proteins, at the biological process level, differentially expressed proteins were significantly enriched in post-translational protein modification, platelet degranulation, regulation of cytokine biosynthetic process, regulation of NIK/NF-kappaB signaling, and positive regulation of peptidyl-tyrosine phosphorylation, among others. At the cellular function level, differentially expressed proteins were significantly enriched in the nuclear lumen, intrinsic component of the membrane, Golgi apparatus, and others. At the molecular function level, differentially expressed proteins were significantly enriched in growth factor activity, enzyme binding, and others. For significantly downregulated proteins, at the biological process level, differentially expressed proteins were significantly enriched in negative regulation of cytokine production. At the cellular function level, differentially expressed proteins were significantly enriched in the nuclear lumen, presynaptic active zone membrane, perinuclear region of cytoplasm, and others. At the molecular function level, differentially expressed proteins were significantly enriched in lactose binding, urate transmembrane transporter activity, fructose transmembrane transporter activity, and others. The top 10 enrichment results are presented in the form of a bar chart (Fig. 11).

### KEGG pathway and enrichment analysis

**Figure 12:**
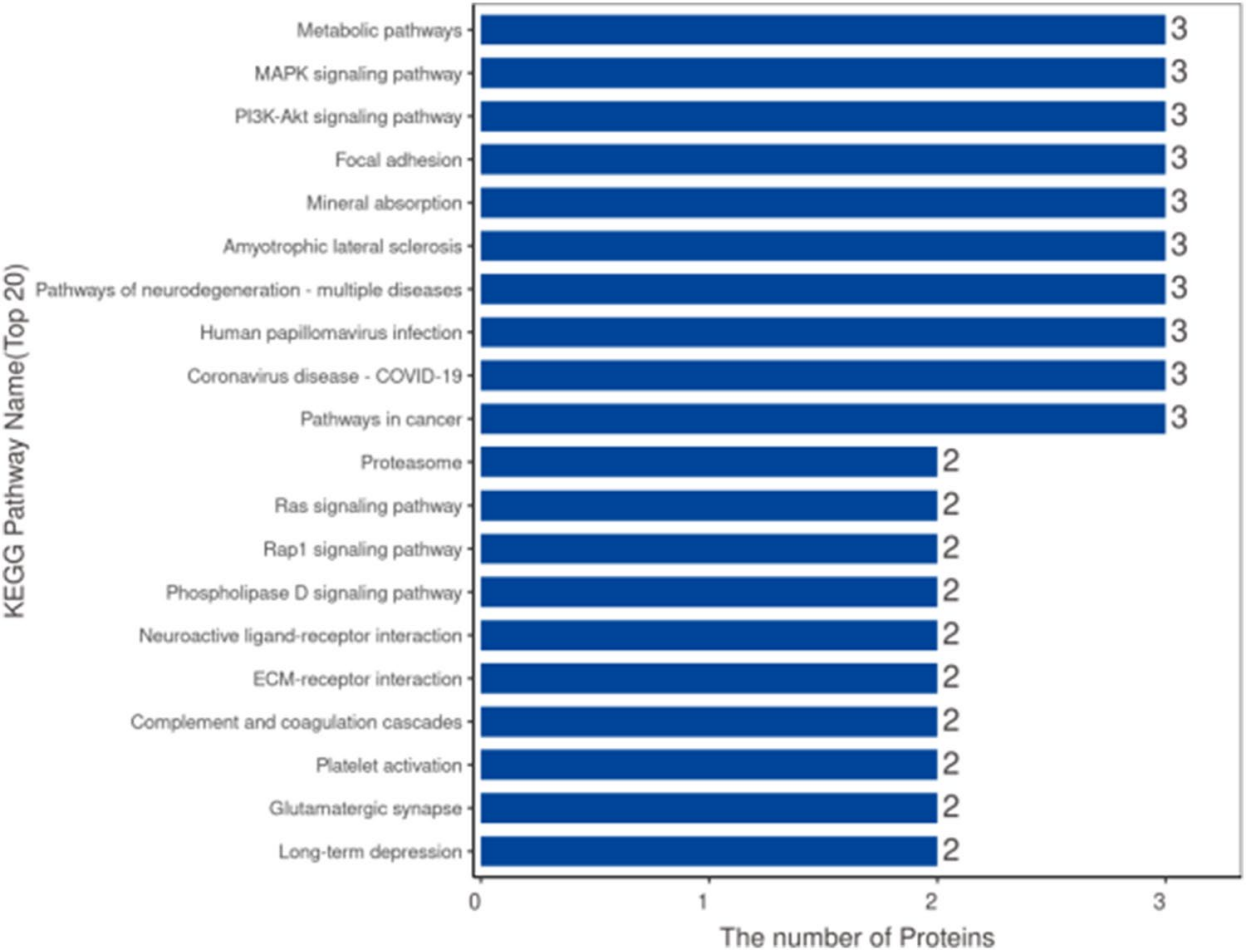
Bar chart of KEGG pathway annotation results (top 20).

We performed KEGG pathway and enrichment analysis on differentially expressed proteins, upregulated proteins, and downregulated proteins. The KEGG pathway analysis results showed that the pathways involved in differentially expressed proteins include metabolic pathways, MAPK signaling pathway, PI3K-Akt signaling pathway, Focal adhesion kinase, and Mineral absorption, among others. The differential protein counts in the pathways were sorted, and the top 20 KEGG pathway annotation results were displayed in a bar chart (Fig. 12).

**Figure 13:**
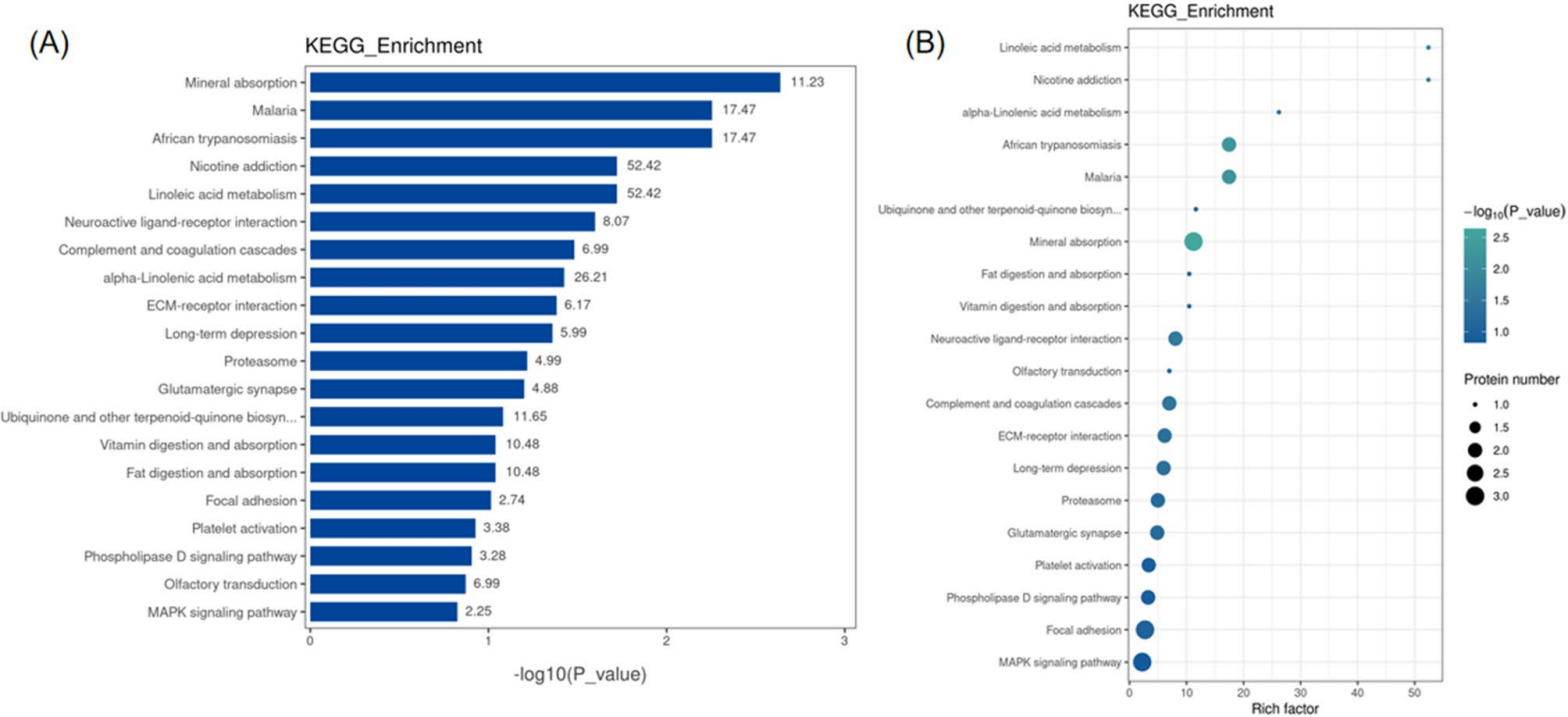
Enrichment analysis of KEGG pathways (top 20). (**A**) Bar chart: Vertical axis represents KEGG pathway names, horizontal axis represents -log10 transformed P-values, and the numbers on the bars represent the Rich Factor values. (**B**) Bubble chart: Vertical axis represents KEGG pathway names, horizontal axis represents Rich Factor values, circle color represents -log10 transformed P-values (darker color indicates smaller P-values), and circle size represents the number of differentially expressed proteins in that pathway.

**Figure 14:**
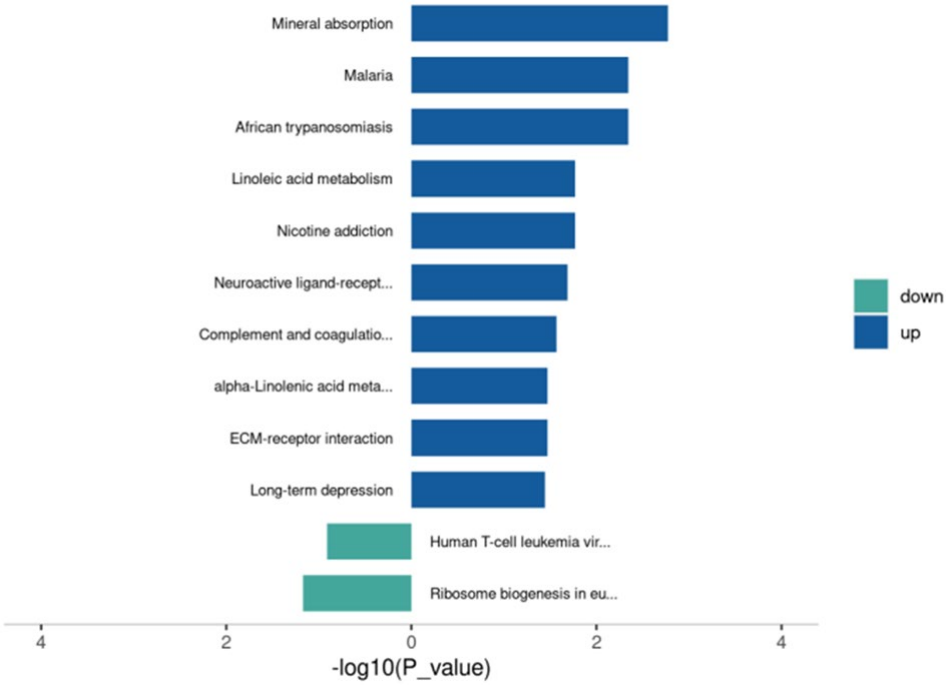
Bar chart of enriched KEGG pathways for upregulated and downregulated proteins. Vertical axis: KEGG pathway names; horizontal axis: -log10 transformed P-values. The longer the bar, the more significant the enrichment. The color of the bar represents the upregulation or downregulation of the protein.

The KEGG enrichment analysis results showed that the overall differentially expressed proteins were enriched in pathways such as Mineral absorption, Linoleic acid metabolism, Neuroactive ligand-receptor interaction, Complement and coagulation cascades, and ECM-receptor interaction. Among them, the significantly upregulated proteins were enriched in pathways such as Mineral absorption, Linoleic acid metabolism, and alpha-Linolenic acid metabolism. The significantly downregulated proteins were enriched in pathways such as Ribosome biogenesis in eukaryotes. The top 20 KEGG enrichment analysis results were displayed in bar and bubble chart formats (Fig. 13). The bar chart of KEGG pathway enrichment for downregulated proteins is shown in Fig. 14.

### Overexpression of miR-373 activated the ERK/MAPK pathway

**Figure 15:**
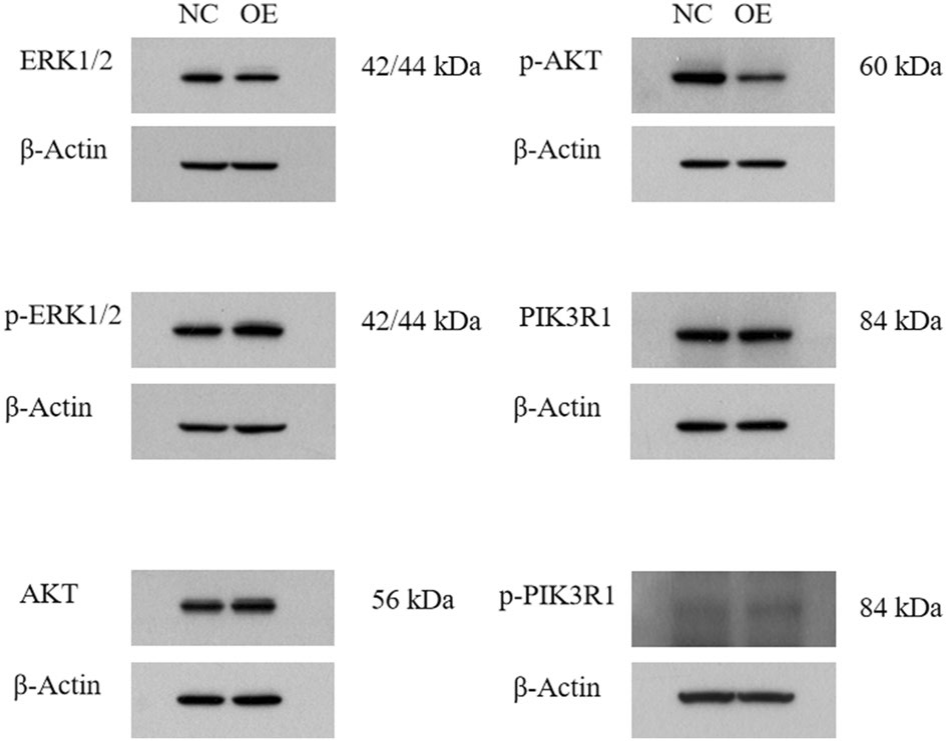
Western blot assay revealed a significantly improved protein abundance of p-ERK1/2 by miR-373 overexpression group as compared with negative control. Protein expression of ERK1/2, p-ERK1/2, AKT, p-AKT, PIK3R1, and p-PIK3R1 in SW480 cells was determined by western blot assays. NC represents the negative control, and OE represents the miR-373 overexpression group. Note: The merged gels produced the resulting image. The original, unprocessed gels and blots are uploaded on the Supplementary Information file. Cropped blots in the body of the paper retain six band widths above and below the band.

Protein and KEGG enrichment analysis results suggested potential involvement in signaling pathways such as the MAPK signaling pathway, PI3K-Akt signaling pathway, and FAK signaling pathway. To validate this hypothesis, SW480 cells were infected with the miR-373 overexpression group and a negative control group, and the expression levels of ERK1/2, p-ERK1/2, AKT, p-AKT, PIK3R1, and p-PIK3R1, which are related to the signaling pathways, were detected using Western blot analysis. The results, shown in the figure below, were analyzed using ImageJ software to calculate the grayscale value of each protein band. The expression level of the NC group was set as 1, and the relative expression level of the target protein in both groups was calculated. The Western blot results showed that overexpression of miR-373 significantly increased the levels of p-ERK1/2 in SW480 cells (Fig. 15). These results indicate that miR-373 may activate the ERK/MAPK signaling pathway to promote invasion and metastasis of colorectal cancer cells.

## Discussion

Despite continuous breakthroughs in early screening and treatment methods, the mortality rate of advanced-stage colorectal cancer remains high. Tumor metastasis is the primary cause of treatment failure and patient death in colorectal cancer. Tumor metastasis is a multi-stage, multifactorial malignant progression process involving abnormal expression and regulation of numerous genes. miR-373 was initially identified as specifically expressed in human embryonic stem cells^15^, and it is involved in the maintenance of stem cells^16^. It has been confirmed that its differential expression is associated with various malignant tumors, and its role in the growth and development of different malignant tumor cells varies. For example, miR-373 plays an anti-cancer role by inhibiting cell proliferation and invasion in breast cancer^17^ and liver cancer^18^. It plays a pro-cancer role in renal cell carcinoma by promoting cell proliferation and inhibiting cell apoptosis^19^. It plays an anti-cancer role by inhibiting cell migration and invasion in lung cancer^20^ and ovarian cancer^21^.

Through overexpression and interference of miR-373 in human colon cancer SW480 cells, it was found that the effects on the cell cycle differed when miR-373 was overexpressed or underexpressed in SW480 cells. However, both overexpression and underexpression of miR-373 could affect the invasion and metastasis of human colon cancer SW480 cells. Overexpression of miR-373 could promote the invasion and metastasis of human colon cancer SW480 cells, which is consistent with the research results of Wang^9^. In addition, interference with the expression of miR-373 could inhibit the invasion and metastasis of human colon cancer SW480 cells, suggesting that miR-373 may exist as an oncogene in human colon cancer SW480 cells. Although this study did not reach a consistent conclusion on the effect of the cell cycle, the disruption of the cell cycle is equally important for the generation of tumor cells. The orderly proliferation and operation of the cell cycle are regulated by the body's regulatory system, while the malignant proliferation of tumor cells is a manifestation of regulatory dysfunction. The cell cycle consists of four phases: G1 phase, S phase, G2 phase, and M phase. Cells in different phases have different physical and chemical properties and characteristics. Changes in the cell cycle mechanism, especially the dysregulation of the expression of cell cycle regulatory proteins that play important roles in tumor development, can lead to the generation of cancer cells. Increasing evidence suggests that miRNAs are closely related to proteins involved in cell cycle regulation^22^. Cell migration and invasion are mainly related to cell skeleton and adhesion. The migration and invasion of cancer cells are important processes in tumor metastasis. Inhibiting the invasion and metastasis of tumor cells is a strategy for inhibiting tumor progression, and multiple approaches can be used for tumor treatment based on this.

Currently, proteomics technology has been widely used in the field of colorectal cancer tumor marker research. For example, Zhang et al. applied LQT-FTMS to analyze the protein expression in 20 pairs of colorectal cancer and adjacent normal tissues and identified 137 differentially expressed proteins. Further validation through Western Blot experiments confirmed that transgelin-2 is a significantly upregulated protein, and its overexpression promotes lymph node and distant metastasis of colorectal cancer^23^. There are few reports on the study of the mechanism of miR-373 in colorectal cancer cells using proteomics methods. This study showed that among the 78 significantly differentially expressed proteins obtained from screening, the GO functional and enrichment analysis results suggested that the differential protein expression products of miR-373 in human colorectal cancer SW480 cells are mainly located in the nuclear interstitium, biological membrane system, Golgi apparatus, etc., and are involved in processes such as post-translational modification of proteins, platelet degranulation, and regulation of cytokine biosynthesis, playing a role in influencing growth factor activity, enzyme binding, and other functions. KEGG pathway analysis and Western Blot results suggested that differentially expressed proteins of miR-373 in human colorectal cancer SW480 cells may be involved in the MAPK signaling pathway. The mitogen-activated protein kinase (MAPK) signaling pathway is an important part of the eukaryotic signal transduction network, involved in regulating cell proliferation, differentiation, and stress response processes^24^. MAPK is a group of evolutionarily conserved serine-threonine kinases, including ERK, p38, JNK, and ERK5. Among them, the Ras-Raf-MAPK signaling pathway, with ERK1 and ERK2 as typical members, is currently a well-studied pathway^25^. miR-373 directly targets the 3'-UTR of mTOR and SIRT1 mRNAs in human fibrosarcoma HT1080 cells, upregulates MMP9 (matrix metalloproteinase 9), and activates the Ras/Raf/MEK/Erk signaling pathway and NF-κB, thereby promoting cell growth^26^. Lan^27^ found in a retrospective study of 1492 cases of stage I-IV colorectal cancer samples that patients with Ras signaling pathway mutations accounted for a higher proportion, reaching 47.3%, and were more prone to lung and peritoneal metastasis compared to wild-type Ras patients. Song^28^ found that the FAK-PI3K/AKT and MAPK pathways may be signaling pathways involved in the inhibition of colon cancer cell proliferation, migration, and invasion by TIMP1. Multiple studies in colorectal cancer have shown that abnormal activation of the MAPK signaling pathway is associated with the occurrence, invasion, and metastasis of colorectal cancer. In summary, miR-373 may regulate the expression of these proteins through a series of mechanisms, thereby affecting the invasion and metastasis process of colorectal cancer cells.

### Supplementary Information

Supplementary Information.

## Data Availability

All data that support the findings of this study are available from the corresponding author upon reasonable request.
