# Supplementary figures and images for: miR-373 promotes invasion and metastasis of colorectal cancer cells via activating ERK/MAPK pathway
Source: Sci Rep. 2024 Jan 2;14:124. doi: 10.1038/s41598-023-49565-5 (PMC10762131; doi:10.1038/s41598-023-49565-5)

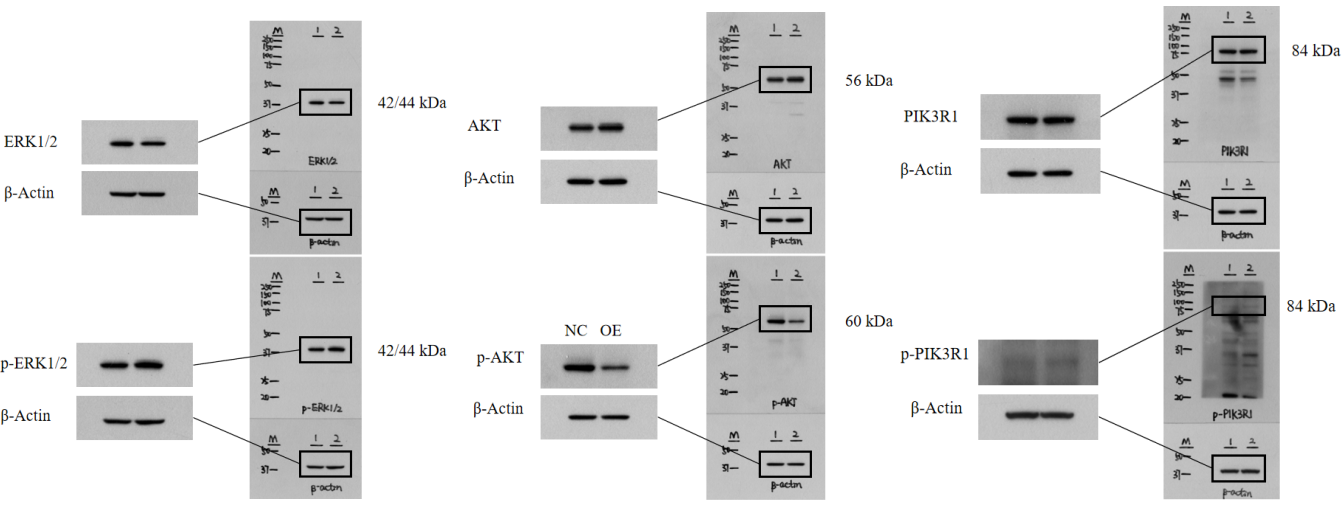

Supplement: Supplementary file 1 — Supplementary Information. [file 41598_2023_49565_MOESM1_ESM.png]
